# Viral deubiquitinating proteases and the promising strategies of their inhibition

**DOI:** 10.1016/j.virusres.2024.199368

**Published:** 2024-04-11

**Authors:** Vera J.E. van Vliet, Anuradha De Silva, Brian L. Mark, Marjolein Kikkert

**Affiliations:** a Department of Medical Microbiology, Leiden University Center of Infectious Diseases (LU-CID), Leiden University Medical Center, Leiden, South Holland, the Netherlands; b The Roslin Institute, University of Edinburgh, Midlothian, Scotland, United Kingdom; c Department of Microbiology, University of Manitoba, Winnipeg, Manitoba, Canada

**Keywords:** Viral deubiquitinating enzyme, Nidoviruses, Innate immune evasion, Antivirals, Ubiquitin variants

## Abstract

Several viruses are now known to code for deubiquitinating proteases in their genomes. Ubiquitination is an essential post-translational modification of cellular substrates involved in many processes in the cell, including in innate immune signalling. This post-translational modification is regulated by the ubiquitin conjugation machinery, as well as various host deubiquitinating enzymes. The conjugation of ubiquitin chains to several innate immune related factors is often needed to induce downstream signalling, shaping the antiviral response. Viral deubiquitinating proteins, besides often having a primary function in the viral replication cycle by cleaving the viral polyprotein, are also able to cleave ubiquitin chains from such host substrates, in that way exerting a function in innate immune evasion. The presence of viral deubiquitinating enzymes has been firmly established for numerous animal-infecting viruses, such as some well-researched and clinically important nidoviruses, and their presence has now been confirmed in several plant viruses as well. Viral proteases in general have long been highlighted as promising drug targets, with a current focus on small molecule inhibitors. In this review, we will discuss the range of viral deubiquitinating proteases known to date, summarise the various avenues explored to inhibit such proteases and discuss novel strategies and models intended to inhibit and study these specific viral enzymes.

## Highlights

• Viral proteases with deubiquitinating activity suggest an antiviral immune evasive effect.
• Antiviral strategies against viral proteases of nidoviruses have been explored extensively.
• Ubiquitin variants show promising inhibition of nidovirus PLpro.

## 1 Introduction

Upon viral infection of a cell, the innate immune response in the host cell is induced through recognition of viral genetic material by pattern-recognition receptors (PRRs). These receptors can, for example, recognise specific viral RNA or DNA species such as double stranded RNA, or other pathogen associated molecular patterns (PAMPs). This then induces the production of type I interferons and inflammatory cytokines in the cell, followed by the production of a number of effector proteins, encoded by interferon stimulated genes (ISGs), in the initially infected cell as well as neighbouring cells, together creating an antiviral state. To date, four groups of PRRs have been identified and acknowledged, namely the Toll-like receptors (TLRs), RIG-I like receptors (RLRs), NOD-like receptors (NLRs) and the HIN-200 family members, with all able to sense both viral RNA and/or DNA (Choubey et al., 2010; Hornung et al., 2009; Kato et al., 2006; Kawai and Akira, 2009; Sabbah et al., 2009; Yoneyama et al., 2004). Besides the well-known post-translational modification (PTM) phosphorylation, another PTM, namely ubiquitination, of many cellular substrates has an important role in innate immune signalling. Ubiquitin is a 76 amino acid protein that is highly conserved in nearly all eukaryotic organisms (Komander and Rape, 2012). Ubiquitination of proteins modifies their function and is critical for regulating most eukaryotic processes (Swatek and Komander, 2016). Multiple research groups have reported this need for ubiquitination of host proteins for activation of the interferon beta (IFN-β)-production pathway. For example, it was found that conjugation of Lys63-linked ubiquitin to retinoic acid-inducible gene I (RIG-I) led to the induction of IFN-β and nuclear factor κB (NF-κB) promoter activity, and it is believed that this conjugation is mediated by tripartite motif-containing protein 25 (TRIM25) and the RING-finger protein REUL at the caspase recruitment domain (CARD)-like region and Riplet at the C-terminal region of RIG-I (Gack et al., 2007; Gao et al., 2009; Oshiumi et al., 2009, 2010). The importance of the ubiquitination of RIG-I was confirmed when it was found that this conjugation was increased upon Sendai virus (SeV) infection (Gack et al., 2007). Furthermore, tumour necrosis factor receptor (TNFR)-associated factor 6, or TRAF6, works as an E3 ligase for Lys63-linked ubiquitination of the IκB kinase to mediate the activation of NFκB (Ning et al., 2008). The authors also showed that TRAF6 is needed for the ubiquitination of interferon regulatory factor 7 (IRF7) through its E3 ligase activity, which in turn produces type I interferons.

Additionally, it was found that the E2 conjugation enzyme Ubc5 plays an important role in the activation of interferon regulatory factor 3 (IRF3) (Zeng et al., 2009). This finding was verified in virus-infected cells, where upon depletion of Ubc5 from the cell, no activation of IRF3 was found, which was restored upon reconstitution of the cells with recombinant Ubc5. This effect can possibly be explained by the need for ubiquitination of the mitochondrial antiviral-signaling protein (MAVS), which others have shown happens upon Sendai virus infection of the cell, as well as upon stimulation with the dsRNA analogue poly I:C (Paz et al., 2009). IRF3 is known to be post-translationally modified in many ways, including by phosphorylation, ubiquitination, sumoylation and ISGylation, in order to induce, maintain, and (down-)regulate IRF3 activation (Shi et al., 2010). For example, upon viral infection, HERC5, an E3 ligase, is recruited to provide the ISGylation of IRF3 and stabilise the protein in the process. This was shown to lead to induction of downstream genes, and thus positively regulating the antiviral responses in the cell.

These findings all demonstrate the importance of ubiquitination and ISGylation of host factors for the activation of the antiviral innate immune response in the cell. Since the down-regulation of the innate immune response also needs to be tightly regulated, several host deubiquitinating enzymes have an important role in negating or inhibiting innate immune signalling. For example, deubiquitinating enzyme A (DUBA) is able to negatively regulate the interferon response induced by PRRs to maintain homeostasis (Kayagaki et al., 2007). Deubiquitinating (DUB) enzymes can recognize the C-terminus of Ub (_73_LRGG_76_) and cleave the isopeptide bond downstream of the terminal Gly_76_ to remove Ub from cellular proteins (Komander and Rape, 2012). Various human DUB families have been identified over the years as part of the ubiquitin system and they belong to either of two classes: the cysteine proteases or the metalloproteases (Amerik and Hochstrasser, 2004; Komander and Rape, 2012; Snyder and Silva, 2021). The three most abundant DUB families are the ubiquitin-specific proteases (USPs, sometimes also called UBP), the ubiquitin carboxy-terminal hydrolase (UCH) and the ovarian tumour (OTU) domain proteases, all part of the cysteine proteases characterised by a catalytic triad consisting of at least an active cysteine residue and a histidine (Amerik and Hochstrasser, 2004).

To evade the innate immune response in the cells, several viruses have evolved to code for similar deubiquitinating enzymes that are able to cleave ubiquitin chains, and/or other ubiquitin-like modifiers such as interferon stimulated gene 15 (ISG15), from cellular substrates. They subsequently curb this antiviral mechanism of the cell to create an environment conducive to viral replication.

## 2 Deubiquitinating proteases of animal viruses

The first mention of such a viral protease with deubiquitinating activity was in 2002 when Balakirev et al. reported the DUB activity of the adenoviral Avp protein (Balakirev Maxim et al., 2002). The authors showed that upon adenovirus infection, an increased deubiquitinating activity could be detected in cells. The Avp protein is a cysteine protease that is essential for viral polyprotein processing and is crucial for mature viral particle formation. Besides establishing its DUB activity, they also showed that the adenoviral Avp protein was able to process pro-ISG15, to create the mature ISG15 protein product. This established the viral Avp protease not only as a protease that can process viral polyproteins, but also as a DUB and a deISGylation enzyme. Subsequently, as few years later, another group predicted the presence of a possible deubiquitinating domain at the N-terminus of the massive L protein of Crimean-Congo Haemorrhagic Fever virus (CCHFV) (Honig et al., 2004). They reasoned that as previously established viral DUBs contained Ovarian Tumour Domain (OTU)-like protease motifs (Makarova et al., 2000) and the zinc-finger-like motifs in this domain were implicated to be responsible for their deubiquitinating activity, CCHF L protein might also have such activities as it harbours an OTU-like domain with a zinc-finger-like motif as well. Somewhat concurrently, Sulea et al. (2005) suggested the possible DUB activity of the SARS-CoV papain-like protease (PLpro). Like Balakirev et al. showed for the adenoviral Avp protease, Sulea et al. already speculated then that viral proteases with DUB-like domains like that of SARS-CoV PLpro, might also be able to deconjugate ISG15, a ubiquitin-like protein. This activity, termed deISGylation, might find its uses in viral innate immune evasion. The DUB activity of SARS-CoV PLpro was eventually confirmed in 2005 by two independent groups, namely Barretto et al. (2005) and Lindner et al. (2005). While the former focused more on the viral polyprotein cleavage by the protease, and only showed the cleavage of di-Ub and the inactivity of the C1651A catalytic mutant (Barretto et al., 2005), the latter did not only show the in vitro cleavage of K48-linked Ub2-7, but also the cleavage of ISG15 by PLpro (Lindner et al., 2005). The structural basis of this deubiquitinating activity was then elucidated by Ratia et al. (2006), who showed clear overall similarities with known host DUB proteins like HAUSP and USP14. An interesting find was that the SARS-CoV PLpro N-terminal domain has a structural fold highly comparable to that of ubiquitin, classifying it as a ubiquitin-like domain, which might play a role in activating the enzyme and dictating its catalytic rate (Leggett et al., 2002), or mediate protein-protein interactions. However, the function of the UBL domain is still largely enigmatic to date (Báez-Santos et al., 2014b; Bosken et al., 2020; Clasman et al., 2017).

Besides the ubiquitin specific protease (USP)-like PLpro of SARS-CoV, of the order *Nidovirales*, Frias-Staheli et al. (2007) reported the deubiquitinating activity of two RNA virus OTU domain-containing proteases from two unrelated viral families. Firstly, as had already been suggested previously, the L protease of CCHFV, of the order *Bunyavirales*, was indeed found to have deubiquitinating and deISGylation activity. These authors were also able to establish that this activity was dependent on the OTU domain of the L protein and that the predicted catalytic site was indeed crucial for this effect. Furthermore, they were able to confirm the DUB and deISGylation activities of the nairovirus Dugbe virus (DUGV) L protein, which is closely related to CCHFV, as well as the proteases of the arteriviruses (order *Nidovirales*) equine arteritis virus (EAV) and porcine respiratory and reproductive syndrome virus (PRRSV), for which the OTU domain is contained within their non-structural protein 2 (nsp2). In addition, the DUB and deISGylation activities of the OTU-domain of CCHFV was later also confirmed using structural studies, and independent groups investigated the binding of this domain to Ub and ISG15, as well as their binding kinetics (Akutsu et al., 2011; James et al., 2011).

## 3 Viral DUB enzymes as promising drug targets

Bergeron et al. (2010) was later able to reproduce the findings that the L protein of CCHFV has deubiquitinating and deISGylation activity, but also found that this activity of the OTU protease is not required for its RNA-dependent RNA polymerase (RdRp) activity. This was done by showing that the OTU domain active-site mutation C40A was not able to hydrolyse Ub-AMC and ISG15-AMC, while its (in vitro) RdRp activity remained intact. Indeed, it was shown that OTU activity of CCHFV RdRp can be blocked independently of its RdRP activity (Tchesnokov et al., 2020). This shows that for some animal viruses, the deconjugation domain is responsible solely for deubiquitinating and deISGylation activity.

For other viruses, however, like those in the order *Nidovirales*, the DUB protease is essential for replication due to its crucial role in cleaving the viral polyprotein. The ability to cleave ubiquitin-like modifiers and affect antiviral innate immune effects, as well as the capability to cleave the viral polyprotein and support replication of the virus, make these DUB proteases promising drug targets. For example, the coronavirus porcine epidemic diarrhoea virus (PEDV) was found to encode a papain-like protease 2 (PLP2) with DUB activity within its non-structural protein 3 (nsp3) (Xing et al., 2013). It was found that not only the overall pool of ubiquitinated proteins was reduced during PEDV infection, PLP2 also specifically deubiquitinates stimulator of interferon gene (STING) and RIG-I upon co-transfection, in turn greatly supressing the IFN-β production. Similarly, porcine reproductive and respiratory syndrome virus (PRRSV) codes for a protease with DUB activity in nsp2, as mentioned prior, which was established to inhibit IFN-β production (Sun et al., 2010). Using a luciferase-based reporter assay, the authors were also able to show that PRRSV nsp2 is able to negatively affect the NFκB activation, which was confirmed when they found that nsp2 was able to block translocation of p65 into the nucleus. Besides these porcine-infecting viruses, the DUB function of the papain-like protease is conserved amongst many others in the order *Nidovirales*. Infection of mouse embryonic fibroblast cells with the *Betacoronavirus* mouse hepatitis virus (MHV) barely induced the levels of type I IFNs, whereas infection of these same cells with Sendai virus, as a control, showed fast and high induction of IFN-β expression (Zheng et al., 2008). Looking further into why this was happening, they found that signal transducer and activator of transcription 1 (STAT1) phosphorylation was undetectable in MHV-A59 infected cells, as well as the absence of IRF3 nuclear translocation. They found that the latter was caused by PLP2-induced deubiquitination of IRF3, as PLP2’s catalytic mutant was not able to cause this deubiquitination. They were later able to determine that MHV PLP2 also exerted its DUB function on TANK-binding kinase 1 (TBK1), a result found using an overexpression system, which also coincided with a lack of IFN-β promoter activation (Wang et al., 2011). Furthermore, this finding was verified by a lack of ubiquitination of TBK1 upon MHV-A59 infection compared to SeV.

**Fig. 1:**
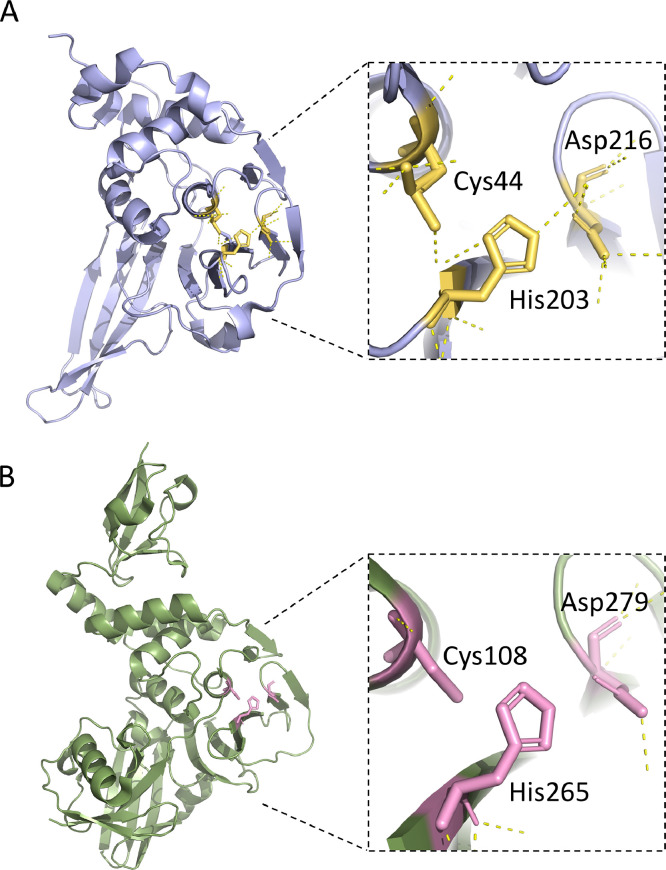
The X-ray structures representing the potential therapeutic targets of active sites in PEDV (A; PDB:6NOZ) and MHV PLP2(B; PDB:5WFI). The close-up view of the catalytic residues of PEDV (Cys44, His203 and Asp216) and MHV (Cys108, His265 and Asp279). The hydrogen bonds are depicted in yellow dotted lines.

The PLP2 proteases of both PEDV and MHV exhibit a conserved structure and catalytic mechanism essential for viral polyprotein processing, making them attractive targets for antiviral drug development. Despite their importance, research on inhibitors targeting PLP2 in PEDV and MHV remains limited. One promising approach is the design of broad-spectrum inhibitors capable of targeting the catalytic triad of PLP2, as depicted in Fig. 1. A recent investigation into the porcine reproductive and respiratory syndrome virus (PRRSV) uncovered that mutations affecting critical residues within PLP2 significantly decrease viral protein production and result in lower viral titres (Bailey-Elkin et al., 2023). This finding suggests the potential efficacy of a similar strategy aimed at targeting PLP2 in PEDV and MHV, specifically focusing on the catalytic residues cysteine (Cys), histidine (His), and aspartate (Asp), could lead to the development of inhibitors with enhanced specificity and potency. Understanding the significance of PLP2 and employing targeted strategies across viral species could pave the way for novel antiviral interventions and vaccine enhancements against PEDV, MHV, and related coronaviruses.

Staying within the order *Nidovirales*, as mentioned before, the SARS-CoV papain-like protease PLpro was found the have DUB and deISGylation activity (Barretto et al., 2005; Lindner et al., 2005). Before these activities were proven, it was already suggested that if the DUB and/or deISGylation activity of SARS-CoV PLpro could be established, besides its ability to cleave the viral polyprotein, this and similar proteases could be highlighted as promising drug targets (Sulea et al., 2005). Even independently, the polyprotein cleavage activity of these proteases makes them promising drug targets. Indeed, SARS-CoV PLpro is essential for polyprotein processing, and thus crucial for replication of the virus (Harcourt et al., 2004; Snijder et al., 2003; Thiel et al., 2003). After the general deubiquitinating and deISGylation effects were also established, multiple groups delved into uncovering the specific sites and activities involved in innate immune invasion. They found that PLpro was able to reduce the ubiquitination of several innate factors like RIG-I, TRAF3, TBK1, STING and IRF3 in cell culture over-expression experiments, and that the protease halted the dimerization and phosphorylation of IRF3, which is normally mediated by STING and TBK1 (Chen et al., 2014). This also resulted in reduced IFN-β production. Another group found the same deubiquitination of RIG-I, TBK1, STING and IRF3 by SARS-CoV PLpro upon co-transfection, as well as establishing that the dimerization of STING was reduced upon SARS-CoV infection (Sun et al., 2012). An overall DUB effect of PLpro was confirmed by Frieman et al. (2009), who also showed that the protease inhibited IFN-β promoter activation after poly I:C and SeV induction, similar to what Devaraj et al. (2007) had shown previously. For a few milder human-infecting coronaviruses, namely HCoV-OC43, HCoV-NL63, HCoV-229E and HCoV-HKU1, the DUB activity of their papain-like protease was more recently uncovered. While the proteolytic and DUB activity of HCoV-NL63’s PLP2 was already discovered in the late 2000s (Chen et al., 2007; Clementz et al., 2010), the DUB and deISGylation activity of the PLP2s from the other common-cold coronaviruses was only firmly established last year (Xiong et al., 2023). It is worth noting that the presence of these proteases in HCoV-229E and HCoV-HKU1, and their importance in viral replication, had already been shown previously (Woo et al., 2005; Ziebuhr et al., 2007). Furthermore, it was shown that the PLPs of the common-cold coronaviruses, as well as the PLpros of the more pathogenic coronaviruses, all had a negative effect on innate immune signaling pathways, presumably caused by its interaction with STING, with the highly pathogenic viruses showing a stronger suppression (Cao et al., 2023; Xiong et al., 2023). This could also possibly correspond to their higher affinity for ISG15 compared to the lesser pathogenic viruses, which show stronger binding to Ub. Altogether, these studies show conserved activities and mechanisms of the papain-like protease across the human-infecting coronaviruses, and may suggest a correlation between varying activities towards Ub and ISG15 and the pathogenicity of coronaviruses.

Another virus that belongs to the order *Nidovirales*, and encodes a comparable papain-like protease with DUB activity, is Middle East respiratory syndrome coronavirus (MERS-CoV) (Bailey-Elkin et al., 2014; Mielech et al., 2014; Yang et al., 2014). By identifying the Ub-binding site on PLpro and subsequent removal of the DUB activity through mutation of this site, it was established that the DUB activity, specifically, was responsible for the inhibition of IFN-β production (Bailey-Elkin et al., 2014). As the catalytic sites on this mutated PLpro were still intact, introduction of such a mutation into the virus enables research into the specific effects of DUB activity upon infection. This concept has since also been explored to create a DUB-negative virus variant that proved to be highly efficient when administered as vaccine candidate (Myeni et al., 2023). Additionally, MERS-CoV PLpro is also known to aid viral replication via proteolytic processing of the viral polyprotein, cleaving the LXGG consensus sites to free nsp1, nsp2, and nsp3 (Yang et al., 2014). Finally, PLpro from MERS coronavirus was found to exhibit deISGylation properties, meaning that it is able to cleave ISG15 from cellular substrates (Mielech et al., 2014). Similar activities were found for SARS-CoV-2 PLpro, including polyprotein cleavage, DUB, and deISGylation activities (Liu et al., 2021; Rut et al., 2020; Shin et al., 2020). Interestingly, despite SARS-CoV and SARS-CoV-2 PLpro being highly identical (Osipiuk et al., 2021), they exhibit different substrate processing preferences. Whereas SARS-CoV PLpro shows the highest activity at cleaving K48-linked ubiquitin and a lower reactivity towards ISG15, SARS-CoV-2 PLpro preferentially cleaves ISG15 from cellular substrates (Shin et al., 2020). Its efficiency at cleaving ubiquitin (both K48 and K63-linked) is notably much weaker (Rut et al., 2020; Shin et al., 2020). As the binding of ISG15 to host proteins is a post-translational modification known to be involved in antiviral immunity, this deISGylation activity suggests another way for the protease to interfere with the innate immune response. Because of these many functions of the viral DUB enzyme, it was established as an extremely promising drug target.

## 4 Plant virus DUBs

**Fig. 2:**
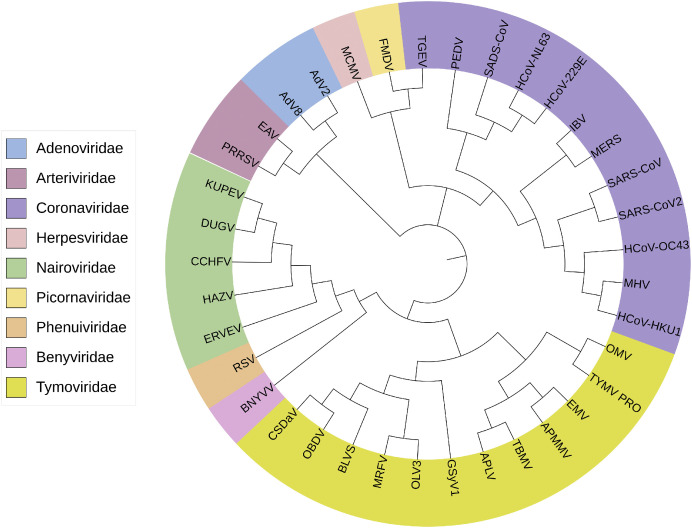
The phylogenetic tree of viral proteases with deubiquitinating activity. The phylogenetic tree was constructed using MEGA 7 software using the maximum likelihood method with 1000 bootstrap replicates. The modifications of the phylogenetic tree were accomplished on the iTOL website, and all the amino acid sequences were obtained from NCBI and PDB websites. The viral DUBs of different viruses are labelled at the tip of the branches, and different colours of branches indicate the different virus families. The arrangement of branches and nodes reflects the inferred evolutionary relationships amongst these viral families. The HCoV-HKU1 is more closely related to the Adenoviridae family of vDUBs, and BNYVV and RSV families may have a common ancestor based on the node of these branches. Adenovirus 2 (AdV2, (Ding et al., 1996), PDB:1AVP), Adenovirus 8 (AdV8, (Mac Sweeney et al., 2014), PDB:4PIQ), Severe acute respiratory syndrome coronavirus (SARS-CoV, (Ratia et al., 2014), PDB:4MM3), Severe acute respiratory syndrome coronavirus 2 (SARS-CoV-2, (Klemm et al., 2020) PDB:6XAA), Human Coronavirus 229E (HCoV-229E, NCBI:NC_002645.1), HCoV-NL63 (NCBI:NC_005831.2), HCoV-OC43 (NCBI:NC_006213.1), HCoV-HKU1 ((Xiong et al., 2023), PDB:7WFC), Middle East respiratory syndrome coronavirus (MERS, (Daczkowski et al., 2017), PDB: 5W8U), Porcine epidemic diarrhoea virus (PEDV, (Durie et al., 2021), PDB:6NOZ), Murine hepatitis virus (MHV, PDB:5WFI), Swine acute diarrhoea syndrome coronavirus (SADS-CoV, (Wang et al., 2020), PDB:6L5T), Transmissible gastroenteritis virus (TGEV, (Wojdyla et al., 2010), PDB:3MP2), Avian infectious bronchitis virus (IBV, (Kong et al., 2015), PDB:4×2Z), Foot- and -mouth disease virus (FMDV, (Swatek et al., 2018), PDB: 6FFA), Murine cytomegalovirus (MCMV, PDB:2J7Q), Equine arteritis virus (EAV, PDB:4IUM), Porcine reproductive respiratory syndrome virus (PRRSV, (Bailey-Elkin et al., 2023), PDB:8EHO), Kupe virus (KUPEV, (Dzimianski et al., 2019), PDB: 6OAR), Hazara virus (HAZV, (Dzimianski et al., 2020), PDB: 7JMS), Dugbe virus (DUGV, (Capodagli et al., 2013), PDB:4HXD), Crimean-Congo haemorrhagic fever virus (CCHFV, (Ekkebus et al., 2013), PDB:3ZNH), Erve virus (ERVEV, (Deaton et al., 2016), PDB:5JZE), Beet necrotic yellow vein virus (BNYVV, NCBI ID:NC_003514), Maize rayado fino virus (MRFV, (Patel et al., 2021), PDB: 7MIA), Blackberry virus S (BLVS, NCBI: NC_038328), Citrus sudden death associated virus (CSDaV, NCBI: NC_006950), Grapevine Syrah virus 1 (GSyV1, NCBI: NC_012484), Oat blue dwarf virus (OBDV, NCBI: NC_001793), Olive latent virus 3 (OLV3, NCBI: NC_013920), Turnip yellow mosaic virus (TYMV, (Fieulaine et al., 2020), PDB: 6YPT), Andean potato latent virus (APLV, NCBI:NC_020470), Andean potato mild mosaic virus (APMMV, NCBI:NC_020471), Eggplant mosaic virus (EMV, NCBI:NC_001480), Okra mosaic virus (OMV, NCBI:NC_009532), Tomato blistering mosaic virus (TBMV, NCBI:NC_021851), Rice stripe tenuivirus (RSV, NCBI ID: NC_003755).

Interestingly, like the DUB proteases from +ssRNA animal viruses, several plant viruses have also been found to code for proteases possessing DUB activity (Fig. 2). As for their animal virus counterparts, plant virus DUBs act as proteases that cleave the viral polyprotein into essential functional protein units during replication (Rodamilans et al., 2018) and also cleave ubiquitin from host proteins to facilitate viral replication by suppressing host antiviral responses (Hua et al., 2018; Lombardi et al., 2013; Wu et al., 2019). Indeed, consistent with animal viruses, plant virus DUBs appear to target host proteins involved in immunity and interfere with pathways that trigger antiviral defences (Chenon et al., 2012). However, unlike our understanding of DUB proteases from animal viruses, much is still to be understood about how these plant virus DUBs manipulate the plant cell to the advantage of the virus. Regardless, the presence of DUBs in both plant and animal viruses conveys the advantage that this enzymatic function must have to virus replication.

**Fig. 3:**
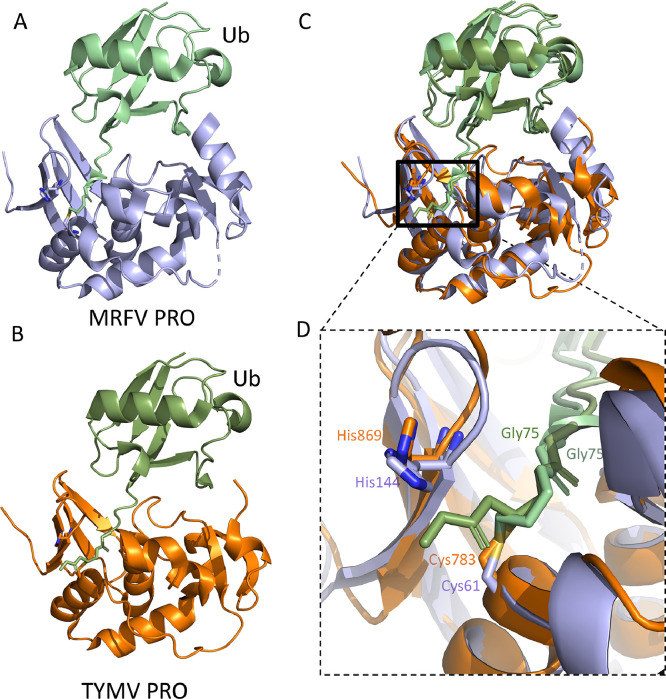
Crystal structure of MRFV PRO bound to Ub and compared with TYMV PRO-Ub. The MRFV PRO is shown in purple (A), and the TYMV PRO is shown in orange (B). The superposition of TYMV PRO-Ub and MRFV PRO-Ub complex is depicted in (C) and catalytic residues of TYMV (Cys783 and His869) and MRFV (Cys61 and His144), as described in (Fieulaine et al., 2020) and (Patel et al., 2021), with the covalent bonds between Gly75 residues of Ub and catalytic Cys-depicted in the close-up view (D).

Of the plant viruses known to encode DUB proteases, those from the *Tymoviridae* family have been best characterized. The *Tymoviridae* family consists of three genera: *Tymovirus, Marafivirus*, and *Maculavirus* (Martelli et al., 2002). Turnip yellow mosaic virus (TYMV) from the genus *Tymovirus* was the first plant virus demonstrated to contain a protease with DUB activity (Fieulaine et al., 2020). TYMV has a monopartite positive-strand RNA genome of 6.3 kb that contains three ORFs. One of them encodes a polyprotein of 206 kDa that contains the methyltransferase (MET), protease (PRO), helicase (HEL) and RNA dependent RNA polymerase (RdRP) domains. The viral PRO enzyme cleaves both the RdRp and HEL from the rest of the viral polyprotein (Jakubiec et al., 2007). Indeed, both proteases are OTU-like deubiquitinating enzymes, and TMYV PRO assists in enhancing viral replication by stabilizing the viral polymerase by removing K48-linked poly-Ub chains from the polymerase to prevent its degradation by the plant ubiquitin-proteasome system (Camborde et al., 2010; Chenon et al., 2012; Jupin et al., 2017). Beyond TYMV, amino acid sequence similarity suggests that the proteases of Andean potato latent virus (APLV), Andean potato mild mosaic virus (APMMV), eggplant mosaic virus (EMV), okra mosaic virus (OMV) and tomato blistering mosaic virus (TBMV) may also possess DUB activity (Fig. 2). A recent study showed that the six *Marafiviruses*, including maize rayado fino virus (MRFV), Blackberry Virus S (BLVS), citrus sudden death-associated virus (CSDaV), Grapevine Syrah virus 1 (GSyV1), oat blue dwarf virus (OBDV) and olive latent virus 3 (OLV3), harbour deubiquitinating activity (Patel et al., 2021). Additionally, the PRO domain of MRFV self-extracts from the viral polyprotein through *cis* cleavage at the PRO|HEL junction. This is in contrast to prior findings in TYMV, where cleavage at the PRO|HEL junction appears to happen in both *cis* and *trans* (Jakubiec et al., 2007; Patel et al., 2021). amongst these plant proteases, only those from TYMV and MRFV have been structurally characterized (Fieulaine et al., 2020; Patel et al., 2021). Unlike the catalytic triad observed for their closest structural homologues within the ovarian tumour domain, MRFV PRO and TYMV PRO only have catalytic dyads (MRFV PRO: Cys61 and His144, TYMV PRO: Cys783 and His869). A superposition of the proteases from TYMV and MRFV bound to Ub reveal similarities that could be exploited for inhibitor designs (Fig. 3). However, delivery and uptake of small molecule inhibitors as an antiviral to protect crops from infection may be challenging. Alternatively, there are potential transgenic approaches that could be explored using protein based vDUB inhibitors as described below.

Though roughly half (∼45%) of all plant viruses encode proteases (Rodamilans et al., 2018), little has been done to exploit them as targets for infection control. Most agronomically essential plant viruses, including *Potyviridae, Tymoviridae* and *Bromoviridae*, employ cysteine proteases as part of their replication and infection mechanism. Thus, these viral proteases could serve as promising antiviral targets; however, delivery of small molecule inhibitors to a field of crops would clearly pose significant challenges. Furthermore, developing and implementing transgenic plants expressing inhibitors could potentially address the issue, but it's crucial to acknowledge that this process may require a significant amount of time. Since developing transgenic plants involve various steps, including genetic engineering, extensive testing, regulatory approval, and widespread adoption by farmers.

## 5 Inhibitors of viral DUB enzymes

Several attempts have been made to generate antivirals targeting DUB enzymes of animal-infecting viruses. One such strategy is the use of proteolysis-targeting chimeras (PROTACs) (Ahmad et al., 2023; Liang et al., 2023). This is based on degradation of the target (viral) protein by directing its polyubiquitination, which will then lead to proteasomal degradation (Liang et al., 2023). Such PROTACs consist of a ligand that is able to bind the (viral) protein of interest, linked to a ligand able to recruit an E3 ligase and induce ubiquitination of the target protein. However, poor solubility and cell permeability of these compounds have been reported, causing problems with bioavailability (Liang et al., 2023; Murgai et al., 2022). Another example is the use of ebselen and its derivatives, which have been shown to have an antiviral effect targeting SARS-CoV-2 PLpro, its main protease Mpro, and nsp14 (Zmudzinski et al., 2023). However, as it inhibits multiple viral proteins and was found to be a general inhibitor of cysteine residues, its specificity for the viral DUB domain is low and could therefore have unwanted effects on the host. In this respect, it may not be a promising drug candidate. In addition, others explored the compound F0213 as a pan-coronaviral PLpro inhibitor, but were not able to determine the structural binding mode despite its effectiveness in cells and animal models (Yuan et al., 2022). According to the authors, this might have been caused by low affinity of the compound to PLpro, indicating possible insufficient binding.

Besides these candidates, most other proposed drugs are small molecular inhibitors that inhibit cellular DUBs. For example, WP1130, a small molecule inhibitor targeting a wide range cellular DUBs like USP5, USP14 and UCH37, exhibits antiviral effects against both murine and human norovirus, underscoring the role of (cellular) DUBs in governing virus replication (Perry et al., 2012). Due to the poor solubility of WP1130, a derivative of this compound, called Compound 9, was tested and substantially decreased murine norovirus replication (Charbonneau et al., 2014). Several derivatives of WP1130 retain their extensive antiviral capacity, effectively targeting a range of RNA viruses, including Sindbis virus and La Crosse virus (Gonzalez-Hernandez et al., 2014). Additionally, the compound C6, featuring a 2-cyano-3-acrylamide structure, contributes to the inhibition of intracellular murine norovirus replication by suppressing DUB activity (Passalacqua et al., 2016). Upregulation of gga-miR-30d reduces the proliferation of infectious bronchitis virus (IBV), with USP47 being identified as a cellular target of gga-miR-30d (Li et al., 2020). The DUB inhibitors P22077 and PR-619, which target cellular DUBs USP7 and USP47, disrupt Gag processing, ultimately impeding the replication of HIV-1 (Setz et al., 2017). However, while illustrating the effectivity of manipulation of DUBs to inhibit virus infections, these are all inhibitors against cellular DUBs, which has the potential of causing adverse effects. As many host DUBs have been identified to be important in cancer biology, for example, inhibiting them could have an unwanted effect on cancer cells or cause other pathologies, as their deregulation is often found to be involved with an array of diseases (Drag and Salvesen, 2010; Farshi et al., 2015; Mirzapoiazova et al., 2020). Therefore, targeting viral DUBs would be a safer strategy.

**Fig. 4:**
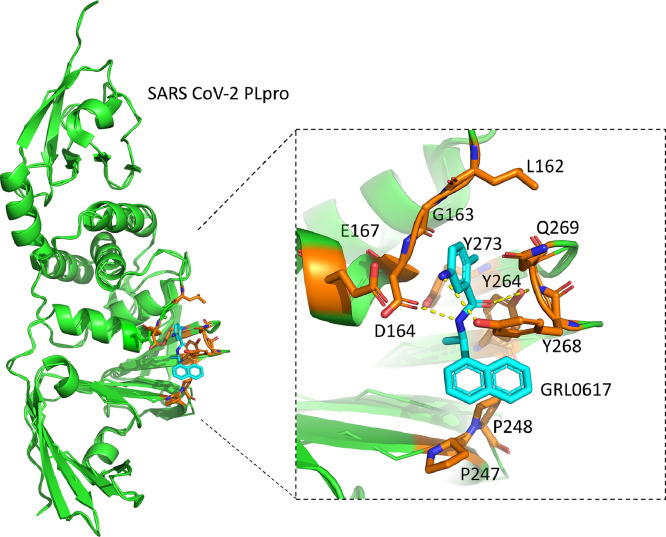
The complex structure of SARS-CoV-2 PLpro with GRL0617 (PDB: 7CJM). The close-up view of interactions between inhibitor GRL0617 (cyan) and surrounding residues of SARS-CoV-2 (Leu162, Gly163, Asp164, Glu167, Pro247, Pro248, Tyr264, Tyr268, Gln269, Tyr273) in orange colour. Hydrogen bonds are depicted in yellow dotted lines.

**Fig. 5:**
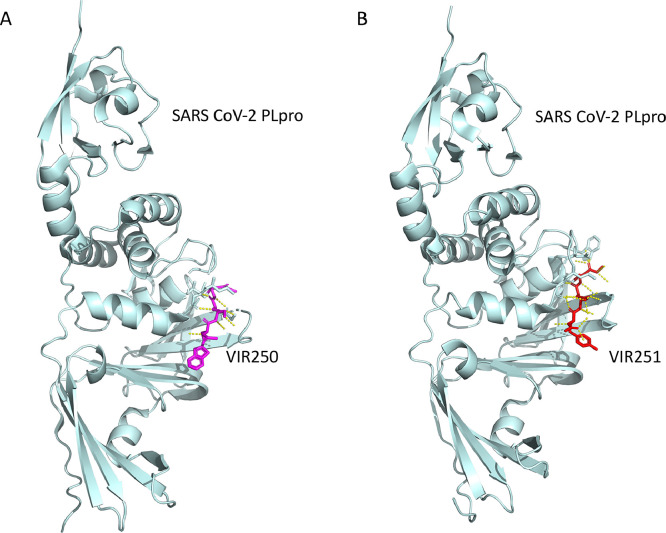
The X-ray crystal structures of VIR250 and VIR251 inhibitors in complex with SARS-CoV-2 PLpro (A and B). SARS-CoV-2 PLpro/VIR250 (PDB: 6WUU) and SARS CoV-2 PLpro/VIR251 (PDB: 6WX4) complexes showed a similar network of interacting residues with predominantly hydrophobic interactions (yellow dotted lines).

An example of this is a mutated version of the human cytomegalovirus (HCMV) UL48 deubiquitinating enzyme, known as the C24 mutant, engineered with mutations in the active site residues that completely abolish DUB function, which leads to a substantial decrease in both HCMV viral protein expression and the proliferation of the virus itself, while still remaining viable (Kim et al., 2009; Wang et al., 2006). This shows that inhibiting the DUB activity of viruses is a promising strategy to obstruct viral infection. Furthermore, other avenues have been researched as well. The 6-mercaptopurine (6MP) and 6-thioguanine (6TG) compounds were initially identified to be used for cancer chemotherapy for the treatment of acute lymphoblastic or myeloblastic leukaemia, but based on computational docking 6MP and 6TG were identified as potential inhibitors of SARS-CoV and MERS-CoV PLpro (Chen et al., 2009; Cheng et al., 2015; Chou et al., 2008). Another remarkable example of a SARS-CoV inhibitor is GRL0617 (Fig. 4), which binds to the PLpro enzyme and neutralizes its DUB function in vitro, consequently hindering viral infection as was demonstrated using a cell viability assay (Ratia et al., 2008). Additionally, an inhibitor based on naphthalene and aimed at SARS-CoV-2 PLpro has been proven to restrain the activity of PLpro, hampering viral replication by interfering with its deubiquitinating activity (Freitas et al., 2020). With the emergence of SARS-CoV-2, much effort has gone into identifying potential SARS-CoV-2 PLpro inhibitors. The possibility of using the above-mentioned compound GRL0617 (and its derivatives) against SARS-CoV-2 PLpro has been revisited (Fu et al., 2021; Ghosh et al., 2023; Osipiuk et al., 2021). Such promising inhibitory candidates initially based on GRL0617, called XR8-23 and XR8-24, were identified and found to have good in vitro efficacy (Shen et al., 2022). Surprisingly, these leads do not bind PLpro at its catalytic site, but in a novel “BL2 groove”. Other derivates of GRL0617 were also researched. One such design found candidates, denoted compounds “7” and “14”, that are more potent than GRL0617 itself at inhibiting SARS-CoV-2 and did bind PLpro at the active site (Sanders et al., 2023). The authors do, however, make note of some liabilities in their compounds like the use of a naphthyl group, which could make the compound prone to timely degradation. A few alternatives for such groups have been suggested but will need further testing and validation. Other tested GRL0617 derivatives are a series of compounds named “Jun”, which showed promising IC_50_ values against SARS-CoV-2 (Jadhav et al., 2024; Ma et al., 2021). They do, however, share the same aforementioned instabilities. Two other new inhibitory compounds, VIR250 and VIR251, were also identified (Fig. 5) (Rut et al., 2020). These two compounds showed great promise as inhibitors against SARS-CoV and SARS-CoV-2 PLpro, and were deemed adequately selective as they were much less effective at inhibiting MERS-CoV PLpro. Unfortunately, none of these compounds or ideas have made it to market (Lv et al., 2022; Ma and Wang, 2022). This might be due to the danger of adverse effects of these small molecular compounds as the coronavirus PLpro enzymes are structurally very similar to human USPs (Ratia et al., 2006). In some studies, inhibitors against SARS-CoV PLpro or SARS-CoV-2 PLpro were screened against a panel of human USPs to rule out potential side effects (Báez-Santos et al., 2014a; Shan et al., 2021). Other studies had a similar approach and tested SARS-CoV-2 PLpro drug candidates against the human protease USP21 to assess the safety of the drugs, where some were found to also inhibit USP21, indicating non-specificity (Calleja et al., 2022; Klemm et al., 2020). For this reason, it is important to identify inhibitors that have high specificity to the viral DUBs, without affecting human DUBs that are structurally alike.

## 6 Ubiquitin variants as inhibitors of viral DUBs

**Fig. 6:**
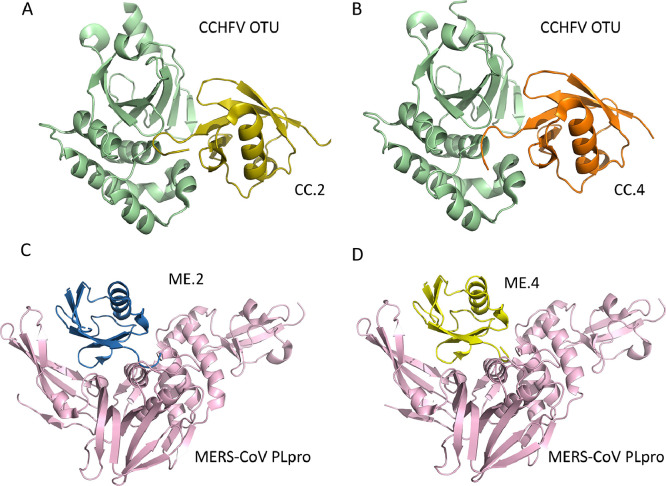
The X-ray crystal structures of ubiquitin variants in complex with CCHFV OTU and MERS-CoV PLpro. The Crystal structure of (A) the CCHFV OTU-CC.2 complex (PDB: 5V5H), (B) the CCHFV OTU-CC.4 complex (5V5G), (C) the MERS-CoV PLpro-ME.2 (PDB: 5V6A), and (D) the MERS-CoV PLpro-ME.4 (PDB: 5V69).

**Fig. 7:**
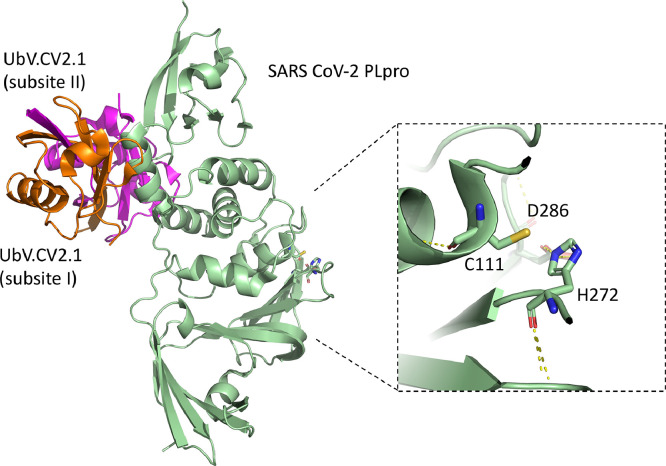
The overall structure of the SARS-CoV-2 PLpro in complex with UbV.CV2.1 (PDB: 8CX9). The complex revealed that UbV.CV2.1 bound to PLpro as an asymmetric strand-swapped dimer. The close-up view of the catalytic residues (C111, His272, Asp286) of SARS-CoV-2 PLpro showed that UbV.CV2.1 bound to a novel site distal to the catalytic site. The hydrogen bonds are depicted in yellow dotted lines.

As has been highlighted above, various viral proteases, including those from the nidoviruses, are able to bind ubiquitin and cleave it from cellular substrates. As an alternative to small molecule-based inhibitors, a novel approach that uses ubiquitin itself as the foundation for protein-based inhibitors against DUB enzymes was developed (Ernst and Sidhu, 2013). Using a phage-display library of ubiquitin amino acid sequence variants (UbVs), it has been possible to identify variants of Ub that potently and specifically inhibit PLpro from MERS-CoV and SARS-CoV-2, as well as the OTU-domain from CCHFV with high affinity (Fig. 6, Fig. 7) (van Vliet et al., 2022; Zhang et al., 2017). These UbVs are mutants of ubiquitin that show remarkably tight binding against the respective PLpros. These studies showed that this novel strategy produced UbV products that were able to inhibit the DUB activity of these viral protases, as well as block the polyprotein processing and viral replication of MERS-CoV and SARS-CoV-2 PLpro in cell culture. Furthermore, the UbV against SARS-CoV-2 PLpro was also able to inhibit its deISGylation activity (van Vliet et al., 2022). These studies showed that the respective UbVs were extremely specific and have a large surface area, in contrast to other antiviral products tested against SARS-CoV-2 PLpro, which are mostly small molecule inhibitors (reviewed in Li et al., 2023). Both of these characteristics of the UbVs reduce the likelihood of off-target effects. Ironically, this specificity also means that the UbVs could not be deployed as a pan-coronaviral drug, as the UbVs against MERS-CoV PLpro do not work against SARS-CoV-2 PLpro and vice versa. Nevertheless, they can be identified rapidly if any new coronavirus arises.

As mentioned, however, these UbVs are protein-based products. This means that further studies are still necessary to establish an efficient manner of delivering these UbVs into cells. As this is not likely to prove feasible in humans soon, it might be useful to look towards possible plant and/or animal models as proof of concept to determine whether such UbVs could work systemically in a complete organism. Additionally, genetic engineering options may be feasible in those species, whereby antiviral UbVs could be expressed from genomic insertions as a way of achieving resistance against important viruses threatening plant crops and animal livestock. Our groups are exploring such avenues now, respectively working to identify UbVs against various plant viruses, as well as the coronavirus avian infectious bronchitis virus (IBV). Using these model systems, we aim to create genetically engineered plants and animals resistant to their respective viral threats. These studies could provide proof of principle that UbV-based DUB inhibitors could work as potent and selective antiviral agents, for which the risk of resistance development in the viruses may be smaller than for regular small molecule antivirals, due to their large binding surface.

## 7 Discussion

Many notable viruses encode a protease with deubiquitinating (DUB) activity that is essential for viral replication. DUBs are found in various virus types, regardless of whether they have RNA or DNA genomes, negative or positive-sense RNA, and infect animals or plants. Most research into viral DUB enzymes have focused on the *Bunyavirales* and the *Nidovirales*, the latter mostly due to the SARS-CoV and the SARS-CoV-2 outbreaks that required immediate action from the scientific community.

These viral proteases serve multiple functions, including polyprotein processing and stabilizing molecules involved in the antiviral immune response through their deubiquitinating activity. In plants, the DUB activity in TYMV protease can protect the RdRP from the ubiquitin-proteasome system by removing the K48-linked polyubiquitin chains (Camborde et al., 2010). Furthermore, the papain-like proteases of the coronaviruses SARS-CoV and human coronavirus NL63 induce the degradation of p53, inhibiting apoptosis and ensuring viral replication in the infected cell (Ma-Lauer et al., 2016; Yuan et al., 2015). Additionally, cellular USP14 has been found to influence the ability of various viruses such as Norovirus, Sindbis virus, Encephalomyocarditis virus and La Crosse virus, to replicate (Perry et al., 2012). These observations suggest possibilities for a common therapeutic approach that can act on various cellular or viral DUBs.

As reviewed here, much research has gone into viral proteases with DUB activity and potential inhibitors that target this activity, mostly based on small molecule compounds. The main challenge in designing these small molecule compounds against viral DUBs is their similarity to host cellular deubiquitinating enzymes, as many such compounds were found to also affect these cellular DUBs. The ability of the virus to mutate and become resistant to small molecule inhibitors is also a challenge. Indeed, structural elucidation of SARS-CoV-2 PLpro with various proposed inhibitors showed many residues under positive selection for antiviral resistance (Sargsyan et al., 2023). The recent discovery of using protein-based UbVs as antiviral strategy is considered to be extremely promising due to the target specificity and potency of the UbVs, as well as their large binding surface, decreasing the chance of the virus evolving to become resistant. However, studying this strategy in plant and animal models might be necessary, and would be beneficial as an initial step to test whether these UbVs have the potential to function effectively in a complete organism, as getting protein products into (human) cells remains an obstacle. There have, however, been new advances in the use of peptides like transactivator of transcription (TAT) that are able to transport protein cargo into cells (Yu et al., 2021). Relatively recently, such a TAT peptide has been used to transport ubiquitin into cells, offering a promising option for UbVs (Hameed et al., 2018).

In conclusion, this review summarises the significance of viral proteases with deubiquitinating (DUB) activity, which are essential for the replication of various viruses, irrespective of their host or genetic characteristics. The potential for using viral DUBs as therapeutic targets becomes evident when considering their capacity to facilitate viral replication, as well as their ability to evade the innate immune response in the cell. Researchers have been exploring potential inhibitors of viral DUB activity, primarily through small molecule compounds. However, the challenge lies in their similarity to cellular DUBs, resulting in unintended effects. Also, there is a need for more extensively validated probe compounds to identify the interactions of viral DUBs with their viral and cellular substrates. This shortage impedes our ability to conduct in-depth research into the biological effects of viral DUB inhibition. However, recent studies suggest that using protein-based UbVs as antiviral tools holds promise due to their target specificity, potency, and reduced risk of viral resistance. Designing these protein-based inhibitors, which can act as selective inhibitors against viral deubiquitinating enzymes, can have great value against emerging coronaviruses in the future.

## CRediT authorship contribution statement

**Vera J.E. van Vliet:** Writing – original draft, Writing – review & editing. **Anuradha De Silva:** Visualization, Writing – original draft, Writing – review & editing. **Brian L. Mark:** Writing – review & editing. **Marjolein Kikkert:** Writing – review & editing.

## Declaration of competing interest

The authors declare that they have no known competing financial interests or personal relationships that could have appeared to influence the work reported in this paper.

## Data Availability

Data will be made available on request.
